# The Difficult Task of Assessing and Interpreting Treatment Deterioration: An Evidence-Based Case Study

**DOI:** 10.3389/fpsyg.2019.01180

**Published:** 2019-07-03

**Authors:** Sarah Bloch-Elkouby, Catherine F. Eubanks, Lauren Knopf, Bernard S. Gorman, J. Christopher Muran

**Affiliations:** ^1^Derner Institute of Advanced Psychological Studies, Adelphi University, Garden City, NY, United States; ^2^Brief Psychotherapy Research Program, Mount Sinai Beth Israel, New York, NY, United States; ^3^Ferkauf School of Psychology, Yeshiva University, New York, NY, United States

**Keywords:** deterioration, treatment failure, case study, outcome, process, patient satisfaction

## Abstract

**Objectives:** Literature on outcome assessment suggests that 35–40% of patients in randomized control trials terminate treatment with unchanged or higher levels of symptomatology. The goal of the present study was to shed light on this phenomenon and the factors accounting for it using a single case study design that investigates the process and outcome of a treatment conducted within a non-randomized clinical trial comparing a cognitive behavioral and a brief relational treatment.

**Method:** The condition of L., a Caucasian man undergoing cognitive-behavioral therapy in a large metropolitan research program, was classified as deteriorating using the Reliable Change Index for the Inventory of Interpersonal Problems (IIP) and the Symptom Checklist-90 (SCL-90). Therapeutic process and outcome were examined using quantitative and qualitative methods rated by several sources.

**Results:** Analysis showed that the treatment was delivered skillfully, and that despite initial difficulties, a strong alliance eventually developed between the patient and the therapist whose perspectives on the outcome of therapy nevertheless diverged. The patient's satisfaction with treatment was high, and he believed his deterioration was caused by its termination.

**Discussion:** Results suggest that the deterioration was not caused by a negative process or a faulty delivery of the therapy. Several explanations were discussed in the context of the literature.

## Introduction

In the last 40 years, psychotherapy outcome research has yielded many large-scale studies examining the process and outcome of various types of psychotherapy and demonstrating its efficacy and effectiveness for a variety of disorders. Treatment failure, including deterioration, defined by Lambert (2011) as a “subset of treatment failure” (p. 414) has yielded far less empirical literature and still remains under-researched. The goal of this study was to investigate this phenomenon at the single case level in order to shed some light on the factors that may be in play and potentially account for deterioration in psychotherapy.

The first impetus to study treatment failure may be traced to a seminal paper by Bergin (1963), in which he hypothesized that in certain cases psychotherapy may fail to facilitate improvement in patients' functioning, and even result in worsening. A series of studies by Lambert and his group subsequently determined that 35–40% of patients in randomized control trials do not improve over the course of therapy, and that among non-responders, 5–10% actually deteriorate, terminating treatment with higher levels of symptomatology (Hansen et al., 2002; Lambert, 2013). Lambert et al.' work further studied therapists' assessment of their patients' outcomes and suggested that therapists are less likely to identify the occurrence of treatment failure than their patients (Lambert et al., 2001; Whipple and Lambert, 2011). They also found that therapists are inclined to rate themselves as above-average clinicians and to underestimate the prevalence of deterioration among their patients (Walfish et al., 2012). Recent meta-analyses reported similar deterioration rates among patients enrolled in internet-based treatments for depression (Ebert et al., 2016; Rozental et al., 2017) as well as among participants in clinical trials for depression and anxiety disorders (Cuijpers et al., 2018).

While these recent studies have established the significant prevalence of deterioration in psychotherapy and called for caution when relying on therapists' viewpoints to assess treatment success, the causes for this phenomenon have not yet been fully determined. In this regard, some studies suggested that client characteristics, such as personality disorders and high levels of comorbidity (Brozovich and Heimberg, 2011), low education (Ebert et al., 2016), high level of interpersonal difficulties, poor motivation, and severity of problem are risk factors for deterioration (Lambert et al., 1977; Mohr, 1995). With regards to the relationship between diagnosis severity and deterioration, research has yielded contradictory findings (see for example Lambert and Bergin, 1994; Lunnen and Ogles, 1998), suggesting that the relationship between diagnosis and deterioration may be limited to pathologies requiring inpatient treatment. Therapist factors, such as lack of empathy, under-estimation of problem severity, negative counter-transference, poor technique, and disagreement with patient about the therapy process, also have been reported as related to deterioration (Mohr, 1995). In a comprehensive review on therapist factors impacting early treatment drop-out, Roos and Werbart (2013) highlighted therapists' skills and experience together with therapists' capacity to provide emotional support and concrete advice. Their work also pointed at the impact of the therapeutic alliance on early termination. Recent work on treatment failure among CBT for specific disorders suggested that some treatments may need to be refined so as to include research advances in areas such as memory and learning (Arch and Craske, 2011). It is reasonable to assert that patient, therapist, and dyadic-relational factors, as well as technical variables associated with the type of interventions chosen and their effective delivery, all combine to determine the treatment success or failure (Boswell et al., 2011).

In response to the scarce literature on treatment failure and deterioration, Dimidjian and Hollon (2010) stressed the importance of further investigating the field. They proposed a comprehensive taxonomy of treatment outcomes that departs from Lambert's conceptualization of deterioration as a sub-category of treatment failure. They noted that psychotherapy outcome cannot be reliably evaluated on the sole basis of symptomatology change, and rather needs to take into account the natural course of the disease a patient would have been expected to go through had they not attended treatment. Accordingly, a treatment may be successful at limiting the rate and the intensity of a naturally deteriorating disease and yet result in unchanged or even worsened symptomatology. In that case, the treatment should be considered successful, despite the patient's worsening. In a similar vein, if a disease is characterized by spontaneous remission, a treatment that would result in a partial remission should be considered unhelpful or even harmful, despite the apparent symptom improvement observed at termination. Dimidjian and Hollon (2011) called for further research on the mechanisms associated with treatment failure and deterioration and promoted the case study approach to investigate these topics. The special issue of Cognitive and Behavioral Practice they edited indeed included rigorous case studies that investigated specific cases of treatment failure and deterioration.

More recent work by Wampold and Imel (2015) further distinguished between treatment deterioration and harm and stated that deterioration can be said to occur when patients' functioning at the end of treatment is poorer than at its start. In contrast, treatment would be considered to have caused harm if the deterioration can be shown to be caused by the treatment itself rather than by factors such as “natural history” (referred to as “natural course of the disease” by Dimdjian and Hollon), life events, or error in measurement (Wampold and Imel, 2015).

An additional layer of complexity in the study of treatment outcome was generated by contradictory findings on the relationship between treatment outcome and patient satisfaction. According to past work (see for example Kazdin, 1977, 1993; Ihilevich and Gleser, 1979), patients with better outcome were found to report higher levels of satisfaction with treatment than patients with comparatively poorer outcomes. In contrast, more recent work suggested that patient satisfaction is not related to therapist- or patient-rated symptomatology change: patients classified as “deterioraters” according to Jacobson and Truax (1991) clinical significance criteria were found to be as likely to be satisfied with their therapy as patients who achieved symptom improvement or recovery (Pekarik and Wolff, 1996; Lunnen and Ogles, 1998). These findings suggest that symptom change may not provide a fully reliable estimate of treatment success, and that additional research is required to determine what constitutes a good or a bad treatment outcome (for further discussion of this question, see Hill et al., 2013; Bloch-Elkouby et al., 2015).

This review emphasized the scarcity of research examining treatment deterioration and the mechanisms that may induce it. This study's goal was to investigate the therapy ingredients potentially responsible for treatment deterioration as well as further clarify the fine line between deterioration and treatment harm. More specifically, this study aimed at assessing whether or not the factors discussed in the treatment failure literature and reviewed in this introduction could indeed be identified as relevant in this case. To this end, we performed an evidence-based case study combining quantitative and qualitative analyses along McLeod's (2011) standards for in-depth single case study analyses.

## Method

### Participants

#### Patient

L. was a 60 year-old Caucasian patient and divorced father of two children. L. lived on his own and has been in a stable relationship with a woman for several years. L. recalled his childhood as having been tainted by a feeling of estrangement from his family as well as his teachers and peers, whom L. experienced as having high expectations he fell from meeting. L. was the son of immigrants from a lower economic status who struggled to provide their children with a better life than they had themselves. L.'s relationship with them was conflicted, as they resented his lack of interest in achievement and what they interpreted as a deficient motivation and effort on his part. In fact, L. did not thrive at school and “never adjusted” to its structure, rather dreaming of becoming a “bohemian artist.” He described himself as a “moody” child, who was temperamental and not easily soothed. When he graduated from high school, L. pursued a college degree as well as some graduate studies that he never completed. L. reported a history of chronic depression and alcohol abuse since his young adulthood. These problems had a negative impact on his marriage and eventually led to the couple's divorce. L. reported that he failed to be an available and attentive father to his children, who grew resentful of his lack of involvement in their childhood. L. did not realize his dream of pursuing an artistic career. He changed jobs several times, struggling to sustain himself, until he finally settled as a computer technician, 16 years prior to starting therapy. L. felt unsuccessful at it his job, and was anxious that he may lose it. Although L. never let go of his old dream of being an artist, he did not undertake any action to accomplish it, either. His current partner was supportive of his artistic aspirations and believed in his ability to finally take action. However, L. felt paralyzed by his procrastination, had little motivation, and felt incapable of moving out of his inertia. L experienced elevated levels of shame and guilt, and constantly compared himself to others, resulting in a pervasive sense of being inferior and defeated. His proclivity toward self-blame alternated with resentment against others for their success and lack of support, resulting in high levels of interpersonal distress and unfulfilling relationships. L did not experience extreme levels of anxiety, but he struggled with constant ruminations about his past failures, with little faith in his capacity to ever change his life path. L. also felt anxious that he was inadequate at his current job and that he might lose it.

L. had been in therapy many times before attending the CBT treatment analyzed in this case study. Between the years 2006 and 2013 he attended therapy several times at the same research program and worked with several therapists in different modalities. At the time of the intake preceding the CBT process examined in this study, L. was in pharmacological treatment and stabilized on Fluoxetine (30 mg per day) and Buspirone (20 mg per day) (i.e., three months at the same dose before starting psychotherapy) to alleviate his depressive symptoms and his anxiety. He also had a prescription for Zolpidem that he used as needed to treat his recurrent insomnia, usually four times per week.

At the intake process, which involved the administration of the Structured Interview for DSM–IV Axis I & II (SCID; First et al., 1995) administered by trained research assistants, L. was not given any diagnosis on DSM-Axis I (American Psychiatric Association, 1994) and only met the criteria for Depressive Personality Disorder on Axis II. More specifically, L. met all the criteria of the Depressive Personality Disorder. He endorsed that his usual mood is “unhappy,” that he sees himself as an “inadequate person,” that he often “puts himself down,” that he is a “worrier,” that he is critical and judgmental toward others,” that he is “pessimistic,” and that “often feels guilty or remorseful” for things he did or did not do. L. did not qualify for alcohol abuse or substance use and reported occasional social drinking (less than once a month). On the target complaints form, L. reported three problems: (1) “I feel torn between being an artist and having a real job;” (2) “I have regrets about having been bad husband and father;” (3) “My present girlfriend feels I am too wrapped up in myself and worry more than I act.”

#### Therapist

L.'s therapist was a Caucasian female therapist in training. L. was the first patient she treated at the research program. She held a Master's degree and was a doctoral student in clinical psychology with 2 years of prior clinical experience. Her training encompassed a psychodynamic and a cognitive-behavioral approach to therapy, but she personally identified with the psychodynamic orientation and had herself been through a psychodynamic psychotherapy. L.'s therapist lived by herself and was involved in a long-term romantic relationship. She did not see herself as affiliated with any religion.

### L.'s Participation in the Research Program

L.'s case was conducted as part of a large psychotherapy research program at Mount Sinai Beth Israel Hospital. The research program involved a clinical trial that compared the process and outcome of a Cognitive Behavioral Treatment for personality disorders (CBT: Turner and Muran, 1992) and a Brief Relational Treatment (BRT; Muran and Safran, 2002). The former is a 30-session long, manualized CBT treatment for personality disorders (CBT: Turner and Muran, 1992) that involved a schema focus (Beck et al., 1990) and a case-formulation framework (Persons, 1989). The latter is a 30-session long treatment based on relational psychoanalysis, humanistic psychotherapy principles (Safran and Muran, 2000) as well as on Muran and Safran's empirical work on alliance ruptures and their resolution throughout treatment (1996). BRT aims at increasing patients' awareness of the relational themes and patterns they are embedded in so as to provide them with the opportunity to reflect on them and change them when desired.

Patients' inclusion criteria to the research program included: (a) 18–65 years old, inclusive; (b) Cluster C personality disorder or personality disorder not otherwise specified (PD NOS) on Axis II of *DSM-IV* (American Psychiatric Association, 1994); (c) willingness to be videotaped; (d) willingness to complete assessment parameters; and (e) English proficiency sufficient to communicate in therapy and complete the questionnaires. Exclusion criteria included: (a) evidence of organic brain syndrome or mental retardation; (b) evidence of psychosis or need for hospitalization; (c) diagnosis of severe major depression (these patients were referred to an outpatient psychiatry service for a combined treatment of CBT with antidepressant medication); (d) diagnosis of bipolar disorder; (e) evidence of active substance abuse; (f) evidence of active Axis III medical diagnosis; (g) history of violent behavior; (h) evidence of active suicidal behavior. Patients stabilized on an antidepressant/anxiolytic medication for 3 months prior to intake were eligible to join the program. After being assessed by trained research assistants, patients who met the inclusion criteria were randomly assigned to the CBT or the BRT condition. Patients committed to stay out of treatment for 6 months following treatment termination, after which they were allowed to apply for another round of therapy if they wished so. Patients who returned to the program underwent the same assessment as newcomers, at the end of which they were, again, assigned to a treatment condition. Patients who met the inclusion criteria but could not join the program immediately due to therapist unavailability were offered to be assigned to one of the conditions on a non-randomized basis. This was the case of L., who did not join the randomized control trial and was rather offered therapy based on therapist's availability.

#### Treatment

The treatment course examined in the present study was L's fourth therapy in the research program, and his first CBT after three utterances of BRT. This treatment was a 30-session long, manualized CBT treatment for personality disorders (CBT: Turner and Muran, 1992) that involved a schema focus (Beck et al., 1990) and a case-formulation framework (Persons, 1989). The treatment entailed two intervention phases: (a) Symptom Reduction, and (b) Schema Change, in which core beliefs were modified or restructured. Both phases included traditional cognitive–behavioral strategies, including self-monitoring, cognitive restructuring, behavioral exercises, and experimentation. The therapeutic relationship was founded on the principle of “collaborative empiricism” (Beck et al., 1979).

L.'s therapist underwent 16 h of didactic training in CBT provided by a licensed professional fellow at the Beck Institute and attended 90-min weekly group supervision. Supervision sessions made use of the videotaped case material and included case formulation, treatment planning, and change strategies. Therapists' adherence to the CBT manual was assessed using a 44-item Likert scale measure of treatment fidelity with demonstrated internal consistency, interrater reliability, and discriminant validity (Santangelo et al., 1994; Patton et al., 1998). L.'s therapist was found to be adherent to the treatment manual.

### Case Selection and Informed Consent

L's case was selected from a dataset of 72 CBT cases that was originally extracted from the global archival data of the research program by the first author to conduct a pilot outcome study assessing the congruence between therapists and patients in the assessment of outcome (Bloch-Elkouby et al., 2015). The 72 cases were extracted according to the following criteria: (1) They completed treatment at the end of the 30-sessions protocol; (2) Their outcome data was complete and included all the patient and therapist-rated measures. Five of these 72 patients reliably deteriorated, as assessed by at least one outcome measure; L. was among the two out of these five who reliably deteriorated on two outcome measures. We chose L.'s case over the other “deteriorator” because more process and video data were available for him than for the other case. L. provided written informed consent for the future presentation and publication of de-identified personal information related to his treatment for research purposes, covering the present case study. The consent authorization form was approved by the Institutional Review Board of Mount Sinai Beth Israel Hospital that houses the research program. In this paper, L.'s identifying demographic information was modified and disguised in order to protect his confidentiality. The letter L does not reflect the patient's true initial and was assigned for ease of reading only.

### Outcome Measures

The assessment battery employed by the study in which L. took part included multi-dimensional measures encompassing symptomatology, interpersonal functioning, chief complaints, and global functioning assessment. The following measures were used:

#### Symptom Checklist-90-Revised

The SCL-90-R (Derogatis, 1983) is a 90-item Likert scale questionnaire (ranging from 0 to 4) which measures nine symptom dimensions and provides three global indices of symptomatology. The Global Severity Index (GSI), used in this study, is obtained by averaging the scores obtained on the 90 items, and is often used as an overall indicator of symptomatology. The measure has shown good internal consistency, ranging from 0.77 to 0.90, and test-retest reliability of 0.84 over a 1-week period (Derogatis, 1983).

#### Inventory of Interpersonal Problems-32

The IIP-32 (Horowitz et al., 2000) is a 32-item Likert scale questionnaire (ranging from 0 to 5) measuring interpersonal functioning. It is composed of 32 items divided into eight scales that add up to a total score. When rated by patients, the measure has shown good internal consistency (Cronbach's alpha is 0.96), and good test-retest reliability of 0.78. Psychometric properties were not reported for the therapist-rated version of the IIP-32.

#### Global Assessment Scale

The GAS (Endicott et al., 1976) is a measure of overall functioning rated by therapists, which includes a 100-point scale divided into 10 equal ranges accompanied with examples of behavior characteristic of the range. No psychometric properties were reported for this measure in the literature.

The SCL-90-R and IIP-32 were completed by L. at intake and at termination. The IIP-32 and GAS were completed by the therapist after the third therapy session as well as at termination.

L.'s change across time was assessed using (Jacobson and Truax, 1991) Reliable Change Index (RCI). RCI was calculated using the test-retest coefficient of the SCL-90 and the IIP-32. Deterioration was operationalized as a reliable worsening exceeding 1.96 SD (Ogles et al., 1995).

### Change Measurement

Reliable change index scores (RCI) were computed according to Jacobson' and Truax' (1991) formula: (Post-treatment scores–Pre-treatment scores)/Sdiff, with Sdiff = standard error of the difference between the two test scores (Sdiff = $\sqrt{}2\left(SE\right)2$, and $\text{SE}=\text{S}1\sqrt{(1}-test-retest$, with S1 = Standard deviation of the measure for the sample examined at intake, and test-retest = test retest reliability coefficient for the measure examined. The RCI scores were transformed into categorical scores following the example set by Jacobson and Truax (1991) and are presented in Table 1.

**Table 1:** Categories of change according to RCI scores.

**Classification**	**Qualitative interpretation**
RCI < −1.96	Reliable improvement
−1.96 < RCI < −0.5	Improvement
−0.5 < RCI <0.5	No reliable change
0.5 < RCI < 1.96	Worsening
RCI > 1.96	Reliable deterioration

### Process Measures

To understand different aspects of the therapeutic process in the present case, several quantitative and qualitative methods were selected to examine fluctuations in the quality of the therapeutic alliance, the impact of sessions, and the client's subjective experience of therapy over time. Additionally, patient-, therapist-, and observer-rated measures were used, so as to provide different perspectives about the therapeutic process and to correct for potential raters' biases.

#### Post-session Questionnaire

The PSQ (Post-Session Questionnaire) (PSQ: Muran et al., 2004) is comprised of several measures evaluating the therapeutic alliance and process by session. The Working Alliance Inventory (WAI; Tracey and Kokotovic, 1989) is a 12-item Likert scale measure assessing the patient-therapist bond and their agreement on tasks and goals, using three discrete subscales which can be combined to yield an average score. The Session Evaluation Questionnaire (SEQ; Stiles, 1980) is a 12-item Likert scale measure assessing the session impact. The measure includes two different subscales: session smoothness (SEQ/S) and session depth (SEQ/D. This study used the overall score yielded by the session's depth subscale as a measure of session impact.

The PSQ also includes three questions about whether a rupture occurred during the session (Rupture Presence), how upsetting it was (Rupture Intensity), and to what degree, if any, it was resolved (Rupture Resolution). Respondents also are invited to provide an open-ended narrative describing the problem (Rupture Description).

L. and his therapist were both required to complete a parallel version of the PSQ after each session. L. was informed that his therapist would not have access to the information he provided on the PSQ. To enforce the confidentiality of L.'s responses, he completed the PSQ in a private area and deposited it in a locked mailbox.

#### Rupture Resolution Rating System

The 3RS (Eubanks et al., 2015, 2019) is an observer-based measure of alliance ruptures and resolution strategies. The 3RS yields ratings for the frequency and significance of withdrawal and confrontation ruptures, as well as the therapist's use of strategies to resolve these ruptures. Ratings are made of 5-min segments, permitting the identification of ruptures and resolution strategies across the course of a session. Comparisons of the 3RS to other methods of identifying alliance ruptures have found that the 3RS is more sensitive (Eubanks et al., 2019). The 3RS also detects more ruptures than methods that identify declines in patient-rated WAI scores (Coutinho et al., 2014). The 3RS has a lso demonstrated predictive validity with respect to dropout (Eubanks et al., 2019). In this study, sessions 1, 5, 15, 25, and 29 (the video for session 30 was unavailable) were initially selected for coding in order to cover the span of the 30-session treatment. In an effort to increase the likelihood of coding a session containing an alliance rupture, the sessions with the lowest patient-rated alliances as measured by the WAI were identified. As the lowest patient-rated session, session 1, had already been selected for coding, the second-lowest session, session 3, was also coded. These six sessions were divided between two pairs of coders, comprised of one doctoral-level psychologist (the second author of this paper and the first author of the 3RS measure) and three graduate students whom the first coder had previously trained to reliability. For this study, coders first coded the sessions independently. Then, each pair of coders met and reached consensus on their ratings. The consensus ratings were used in the data analyses. Coders assigned scores for each type of withdrawal marker, confrontation marker, and resolution strategy: A score of 1 was given in a 5-min segment if the marker was observed; a score of 0.5 was assigned if a weak or somewhat unclear example of the marker was observed. The scores were summed for each session, and mean scores and standard deviations were calculated across the six sessions.

In addition, 21 sessions (70%) were randomly selected for viewing and descriptive analysis.

#### Patient Termination Relationship Interview

Information about the patient's global experience of and satisfaction with therapy and the relationship with the therapist was gathered using the patient termination relationship interview, a semi-structured interview administered by a research assistant at termination to assess the patient's subjective experience of the relationship and the treatment's impact.

## Results

### Therapy Outcome

L. reliably deteriorated on the self-rated version of the IIP-32 and the SCL-90-R throughout the cognitive-behavioral treatment examined in the present study (Table 2). By contrast, and as can be seen in Table 2, the RCI obtained for the scores provided by his therapist about his interpersonal problems suggest some improvement, though not significant enough to be considered reliable. In a similar vein, L.'s therapist gave L. a GAS score of 70 at session 3, and 72 at termination, suggesting that the therapist did not see L. as severely ill at the beginning of treatment, and believed L.'s general functioning was minimally improved by the end of treatment. It is interesting to compare these results to those obtained at the end of the other treatments L. attended at the same research program (Table 2). First, it may be noted that the first time L. received treatment, both he and his therapist agreed that the therapy did not yield any reliable change. This was the only time the three outcome measures converged. Upon termination of the second treatment, however, L. reported reliable improvement in his interpersonal relationships, a conclusion somewhat corroborated by his therapist's report of some improvement, but he also described his symptoms as worsening. When L. ended his third round of therapy, his therapist believed L. had made reliable progress in interpersonal functioning but L. himself did not agree with this assessment. He was equally pessimistic about his symptoms, which he reported as unchanged. As mentioned above, during the CBT treatment examined in the present study, L. reliably deteriorated on the two self-report measures for the first time. Interestingly, his therapist did not concur with this evaluation, and endorsed that L. improved, though not reliably, in interpersonal functioning. Six months after the end of the therapy examined in the present study, L. repeated therapy one additional and last time at the research program. That time, both he and his therapist judged his interpersonal functioning as reliably deteriorated.

**Table 2:** L.'s successive therapy outcomes at the research program.

**Treatment modality**	**IIP rated by therapist**	**IIP rated by client**	**SCL-90**
BRT	No change (RCI = 0.17)	No change (RCI = −0.03)	No change (RCI = −0.11)
BRT	Improved (RCI = −0.90)	Reliably improved (RCI = −2.09)	Worsened (RCI = 1.22)
BRT	Reliably improved (RCI = −2.81)	Worsened (RCI = 0.63)	No change (RCI = 0.22)
CBT	Improved (RCI = −0.82)	Reliably deteriorated (RCI = 2.81)	Reliably deteriorated (RCI = 2.31)
BRT	Reliably deteriorated RCI = 2.81	Reliably deteriorated (RCI = 4.26)	Missing data

*BRT, Brief Relational Treatment (Muran and Safran, 2002)*.

L.'s successive treatments outcome scores seem to indicate a general tendency toward a greater deterioration starting during the treatment examined in the present study (4th treatment at the research program). It may be necessary to mention that all the treatments received by L. at the research program, at the exclusion of the one presented in the present study, followed a relational orientation. In order to make sense of these findings, we will now turn to the therapy process which characterized L.'s treatment.

### Therapy Process

#### Case Conceptualization

The therapist's case conceptualization of L.'s challenges followed Persons (1989), in which the patient's chief complaint is broken down into a list of problems, a proposed underlying mechanism, precipitants, and origins of the underlying mechanisms in the early life.

In L.'s case, the therapist did not document her case conceptualization in the patient file. Based on the video-recordings of her sessions with L., it seems like the therapist initially helped L. break down the chief complaints and therapy goals into more specific problems that could be targeted first. L. was able to follow her lead and focused on his difficulty feeling adequate at work, his fear of losing his job, his procrastination with art, and his tendency to ruminate over the past rather than take action.

L. and his therapist collaboratively explored the precipitants, and found that these problems typically emerged in situations that required L. to take initiative and perform tasks that did not include a clear course of action, or that belonged to areas in which L. was lacking skills. In these instances, he experienced automatic thoughts such as “I am inadequate,” “I brought it on myself because of my bad choices in the past,” “I'm not good at anything,” “This is too hard” or “I am going to lose my job and will not have any money.” These thoughts increased L.'s anxiety level and lowered his mood, which in turn reinforced the self-blame, thoughts of inadequacy, and catastrophic thoughts about his future. In these situations, L. typically resorted to avoiding the tasks he feared and engaged in increased rumination about the past.

Several assumptions typically triggered L.'s automatic thoughts when he faced challenging tasks: “If I don't know how to do this I am nothing,” “If I can't even do this people are going to think I am a loser,” or “If I had done better choices in the past I would know how to do this.” These assumptions were fed by self-schemas characterized by a belief of inadequacy, lacking self-agency, incapacity to meet society's expectations, as well as by schemas of others as rejecting, teasing, disappointed, and incapable of loving or accepting him with his flaws.

L.'s schemas likely originated in his difficulty “fitting in” as a child, as well as in his impression that he was a disappointment for his family, teachers, and surrounding social circles. L. struggled academically and socially throughout his childhood, adolescence, and adulthood, making it reasonable to conjecture that he may have had learning and executive functioning difficulties, and may have met the criteria for a developmental disorder. Feeling different, being incapable of succeeding since his childhood, and experiencing his environment as rejecting likely contributed to his core schemas and their activation anytime he was required to perform a task that he did not have mastery over, regardless of the actual difficulty involved in it.

#### Descriptive Analysis of the Therapy Process

A careful examination of the videotaped sessions suggests that during the initial phase of therapy, from session 1 to 5, L. and his therapist seemed to struggle with establishing a collaborative alliance. During these first sessions, L.'s therapist explained the principles of CBT and the dyad agreed that the treatment's goal would be to help L. identify and modify his maladaptive thoughts and beliefs that trigger his sense of inferiority and render it difficult for him to accomplish his projects. Despite their explicit agreement on tasks and goals and their collaborative work toward the identification of L.'s automatic thoughts, the dyad seemed to have difficulties making a connection and progressing. This difficulty seemed to stem from L.'s proclivity to be passive and distracted, coupled with his therapist's overly directional style. L.'s therapist was in fact a young, energetic, empathic, and hard-working therapist, who seemed to put forth a significant effort to provide a high quality therapy. She seemed to be competent and comfortable with the principles of CBT. By contrast, L. presented as a low-energy depressed patient with flat affect, who was prone to digressions and spent extended periods narrating stories from the past or the present with no clear purpose. L.'s therapist seemed to experience difficulty engaging L. and often redirected him to the task at hand. She also tended to fill in the gaps, and to offer multiple choice answers to L. rather than letting him answer her questions, as if trying to prevent him from getting distracted by his own thoughts. At times, her hard work seemed to impede her ability to be present in the moment and to remain attuned to L. For example, she continuously took notes, often at the expense of maintaining eye contact with L. The more structured the therapist became, the less engaged was L., who seemed to be disinterested in actively working with his therapist in a structured way. Accordingly, his responses became more avoidant, and he superficially complied with her requests. The following vignette illustrates the type of interaction which took place between L. and his therapist between sessions 1 and 5.

“L.: Reading it reinforced this feeling that I was a bad father and a bad husband.

Therapist: ok (pauses, looks at her notebook) and is there anything else as far as [coming to treatment]?

L.: Well I have a girlfriend and–

Therapist (interrupting): It's true!

L.: –and that's you know, that's going well right now–

Therapist: oh great!

L.: …and (pauses)

Therapist: Are you guys living together?

L.: Sorry?

Therapist: Are you guys living together?

L.: No, ehm no.

Therapist: How long have you been together?

L.: Ehm, well 12–13 years.

Therapist: Ok.

L.: And ehm (pauses to think)

Therapist: It's going well.

L.: Yeah yeah. She's, ehm…

Therapist: I read that she's feeling that you're wrapped up. You think, you worry more than you act.”

An examination of the process after session 6, though, suggests some change. If L.'s therapist did not offer different types of interventions, she demonstrated greater flexibility and less directiveness, which seemed to allow for a more active participation on L.'s part. L. indeed became more dominant and contributed more to the sessions, even if he maintained an avoidant and digressive style. The following vignette from session 6 illustrates this change:

“Therapist: That's interesting, why do you think she would, why would she… (pauses). Well part of me wants to investigate and talk more about your girlfriend but she's just not here so it's weird, we can't really…

L.: Right.

Therapist: So I'm more interested in you and your thoughts and your behaviors and how, you know, we can work on that.

L.: Well, how would I want to?

Therapist: That's–

L.: How would I want to handle this?

Therapist: Yeah, how?

L.: I can neither get dragged down into her stuff nor can I just walk away from it.

Therapist: Hmm (pauses).

L.: So how do I want to handle it?

Therapist: Yes, what is the ideal, the best case scenario?”

As therapy progressed, L.'s therapist maintained this more flexible stance, even though she continued to adhere to CBT, its principles, and its structure. She also invited L. to provide feedback about the therapy, and to voice his disagreements and/or unmet expectations as he experienced them.

#### Descriptive Analysis of the Therapeutic Interventions

After L. and his therapist broke down L.'s chief complaints into specific problems, L.'s therapist work focused on helping L. gain awareness of the situations in which these problems emerged as well as the mechanisms potentially accounting for them. More specifically, L.'s therapist helped him increase his capacity to reflect on the negative automatic thoughts that emerged every time L. faced a new or bureaucratic task and uncertainty. Most often, these situations involved requests from L.'s employer to perform tasks L. did not know, administrative chores (such as making appointments or complete paperwork), and L.'s desire to promote his art. L.'s therapist also worked on increasing L.'s capacity to reflect on his subtle mood changes as they occurred, and to use them as indicators that some automatic thoughts had just been triggered by an internal or external stimulus. The therapist then proceeded to challenge the automatic thoughts to identify the assumptions and core beliefs underlying them, and to find more adaptive ones. L.'s therapist also engaged with him in problem-solving, as she typically examined with him life situations and encouraged him to seek alternative and more active behaviors to handle the challenging situations. In these instances, she helped L. break down the tasks that he avoided into smaller easier subtasks that were less anxiety provoking, and worked with him on adequate planning to reduce his procrastination. In the sessions, the cognitive work seemed to have a regulatory effect on L., in that it helped L. face his anxiety rather than avoid it and engage in digressive thinking which eventually increased his sense of being overwhelmed and his anxiety. In this context, the therapist's efforts seemed to contain L. and to alleviate his anxiety during the sessions. It seems, though, that L. did not acquire, throughout the treatment, the ability to structure his thinking in a similar vein. Rather, he typically brought to the sessions a lot of written material about the week's events and interactions, and relied on his therapist to organize it. If his compliance with the therapist's homework assignments speaks to his therapist's success in engaging him, the disorganized quality of the written material also show that L. did not actually learn to organize his thoughts. The same observation can be made with regard to L.'s problem-solving skills. L. was very cooperative with his therapist, and in fact, engaged in all the behaviors she prescribed outside of the sessions despite his well ingrained passivity and proclivity to procrastinate. Yet L. did not initiate problem-solving on his own and did not seem to develop a sense of self-worth and agency on the basis of his successes.

#### L.'s Increasing Anxiety at the End of Treatment

Toward the end of the therapy, starting at session 25, L. started to report increased levels of anxiety. His therapist carefully inquired about it and tried to help L. utilize the cognitive work they learnt throughout therapy. L. was indeed able to apply the cognitive principles during the sessions, but continued to report an increase in anxiety and depression. Per L.'s report, life circumstances triggered this worsening, as he faced changes at work, where he was required to change some of his working methods. For L., who always had difficulties with transitions, the change, together with his fear of losing his job, provoked anxiety and depression. Additionally, in the last therapy sessions L. started to express his anxiety that aging might render it more difficult for him to carry on in his job. The same thought, according to L., led his best friend to commit suicide, and therefore triggered increased levels of anxiety as well other feelings of sadness and mourning for L. This friend used to draw portraits in the street for his living and committed suicide when his physical condition deteriorated and prevented him from continuing this activity. Additionally, L. reported concerns about his girlfriend's health and was worried about the reemergence of a past illness. This increased his concerns about his own health as well as his and his girlfriend's finances.

#### WAI Ratings and Alliance Ruptures

In order to gain an additional perspective on L. and his therapist's dyadic interactional style, we examined L.'s and his therapist's ratings of the therapeutic alliance on the WAI, as well as their report of ruptures.

**Figure 1:**
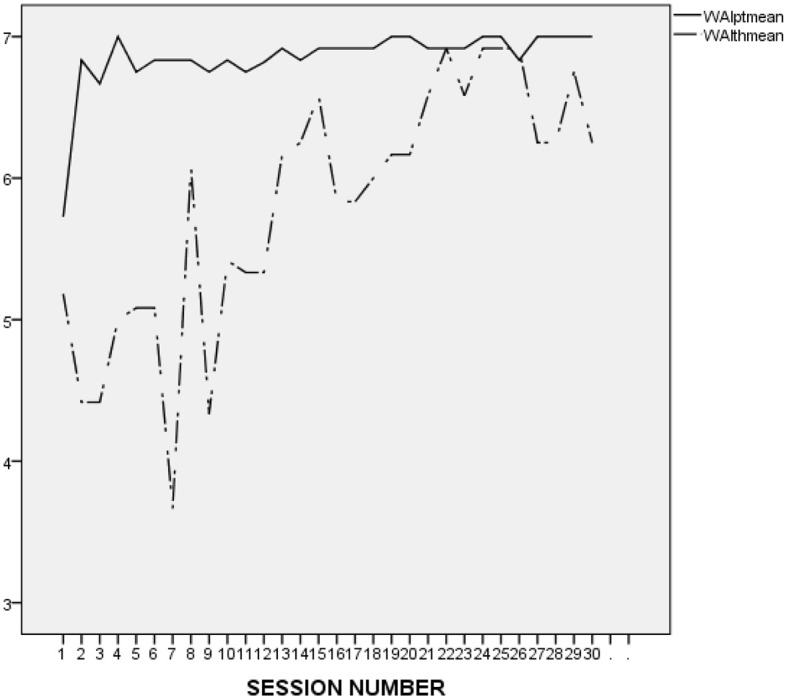
L.'s and his therapist's ratings on the WAI-12 in the course of the treatment investigated in the present study (treatment 4). WAIptmean and WAIthmean respectively, represent the average score on the patient-rated and the therapist-rated Working Alliance Inventory (WAI; Tracey and Kokotovic, 1989) after each session.

As presented in Figure 1, both L. and his therapist seemed to struggle to form an alliance at the beginning of the treatment. It is striking to notice, though, that after the first three sessions, L.'s ratings became flat and almost perfect, as opposed to those of his therapist, which were less inflated and reflected some fluctuation, with a clear tendency toward improvement. In addition, it is worth noticing that L. did not report a single rupture throughout treatment, and that his therapist reported only 3 ruptures, at sessions 1, 2, and 3. L.'s therapist did not provide any narrative to specify the type of rupture that occurred at session 1. At session 2, however, she provided the following description: “patient went on tangents and I did not keep him on task,” which seems to confirm that L.'s therapist experienced L.'s digressive and avoidant style as an obstacle to the establishment of a collaborative working alliance. At session 3, the rupture narrative stated: “patient often went off topic and had to be redirected toward agenda.” The therapist also reported that the rupture was repaired using the following strategy: “I was direct with the patient about our goal for the session and at the same time empathetic to his needs. Suggested he does a thought record on his friend whom he continues to bring up.” This description suggests that L.'s therapist's strategy to repair the rupture was to redirect L. rather than acknowledging the rupture and exploring the possible reasons behind it.

**Figure 2:**
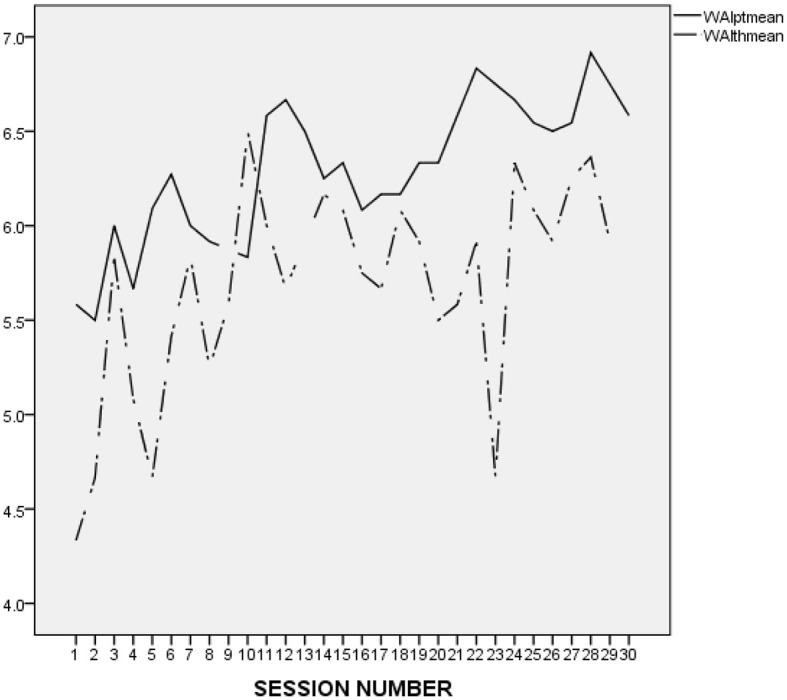
L.'s and his therapist's ratings on the WAI-12 in the course of the 1st treatment at the research program. WAIptmean and WAIthmean respectively, represent the average score on the patient-rated and the therapist-rated Working Alliance Inventory (WAI; Tracey and Kokotovic, 1989) after each session.

**Figure 3:**
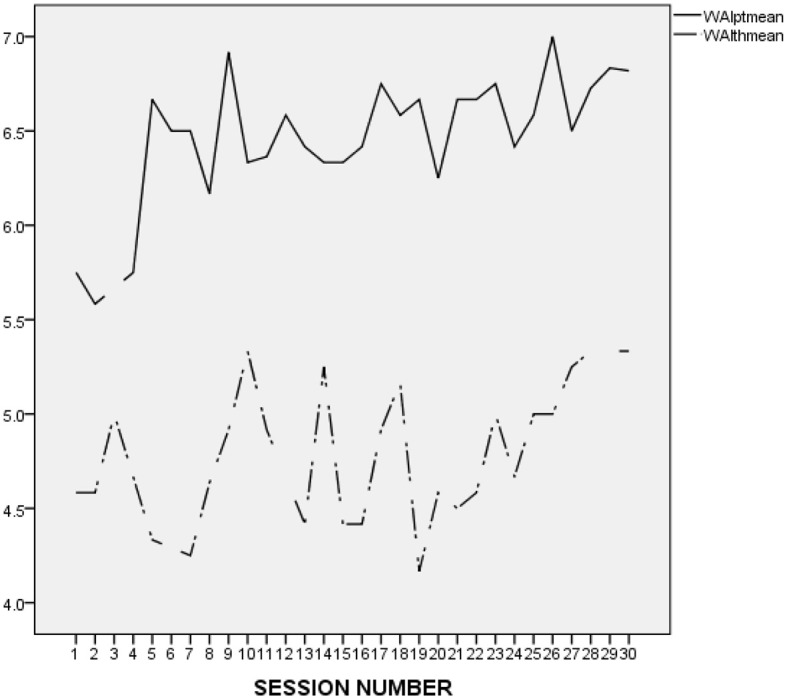
L.'s and his therapist's ratings on the WAI-12 throughout the 2nd treatment at the research program. WAIptmean and WAIthmean respectively, represent the average score on the patient-rated and the therapist-rated Working Alliance Inventory (WAI; Tracey and Kokotovic, 1989) after each session.

**Figure 4:**
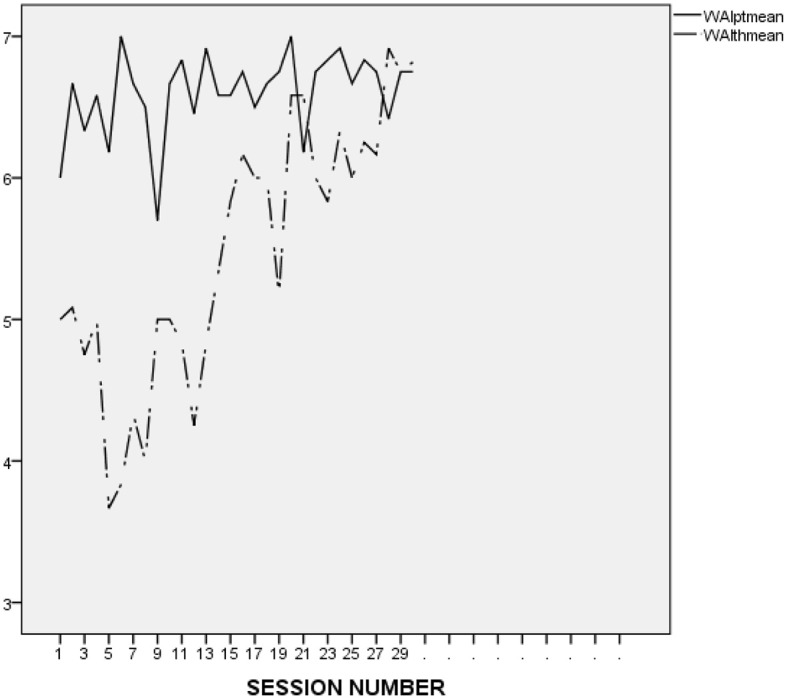
L.'s and his therapist's ratings on the WAI-12 throughout the 3rd treatment at the research program. WAIptmean and WAIthmean respectively, represent the average score on the patient-rated and the therapist-rated Working Alliance Inventory (WAI; Tracey and Kokotovic, 1989) after each session.

**Figure 5:**
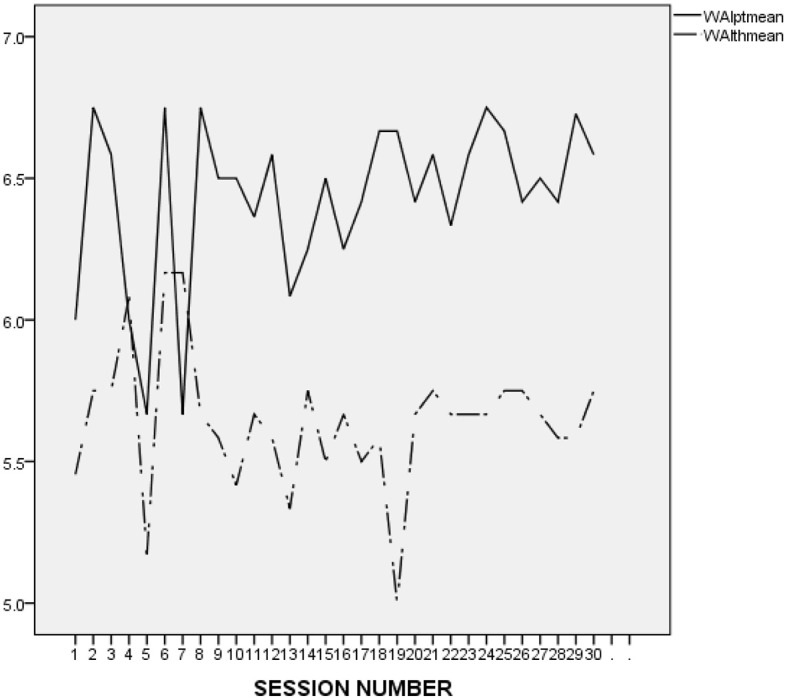
L.'s and his therapist's ratings on the WAI-12 throughout the 5th and last treatment at the research program. WAIptmean and WAIthmean respectively, represent the average score on the patient-rated and the therapist-rated Working Alliance Inventory (WAI; Tracey and Kokotovic, 1989) after each session.

It is difficult to make an accurate interpretation of L.'s linear and perfect ratings. On the one hand, it seems that his ratings were inflated and possibly reflected his inclination to avoid acknowledging conflicts and painful feelings. However, it may be important to compare L.'s alliance ratings to those he provided in the context of the other treatments he attended at the research program. As presented in Figure 2 (first treatment), Figure 3 (second treatment), Figure 4 (third treatment), and Figure 5 (last treatment), these ratings were not as high, and included more fluctuations. This finding suggests that L. may have felt genuinely connected to his therapist throughout his treatment, and developed a stronger alliance with her than with his previous and subsequent therapists.

#### Rupture Resolution Rating System

The most frequent rupture markers were examples of *avoidant storytelling/topic shift* (*M* = 2.33, *SD* = 1.17), which is a form of withdrawal in which the patient tells stories and/or shifts the topic in a manner that functions to avoid the work of therapy. Confrontation rupture markers were less common, but were still evident: the patient expressed some *complaints/concerns about the activities of therapy* (*M* = 0.67, *SD* = 0.88), and there were also instances of *patient defends self against therapist* (*M* = 0.42, *SD* = 0.66), in which the patient defended his thoughts, feelings, or behavior against what he perceived to be the therapist's criticism or judgment. The therapist responded to the ruptures primarily by utilizing the resolution strategy of *inviting the patient to discuss thoughts or feelings with respect to the therapist or the therapy* (*M* = 0.92, *SD* = 0.66), or by *changing the task of therapy* in an effort to re-engage the patient in the work of therapy (*M* = 0.58, *SD* = 1.02).

#### Session Impact

**Figure 6:**
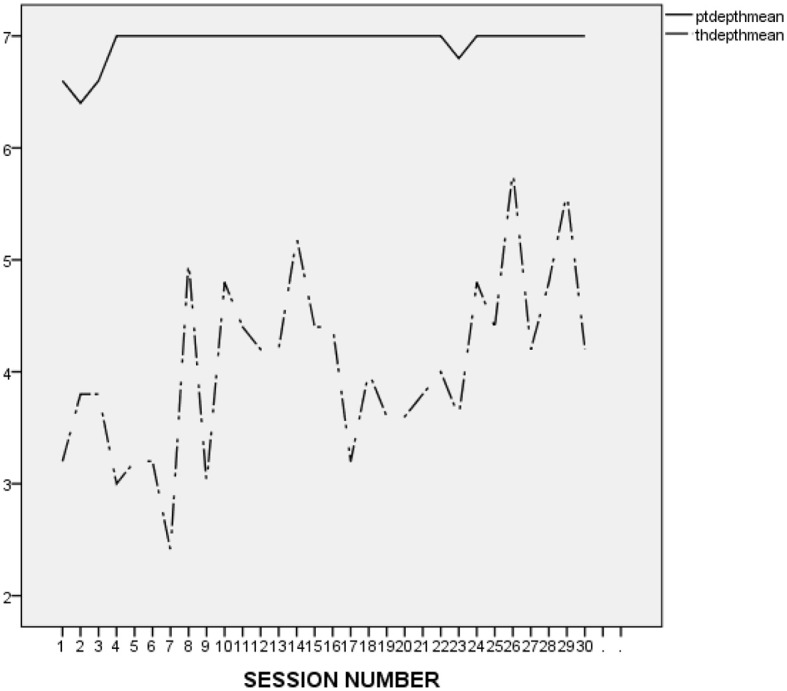
L.'s and his therapist's ratings on the SEQ-Depth in the course of the treatment investigated in this study (4th treatment at the research program). WAIptmean and WAIthmean respectively, represent the average score on the patient-rated and the therapist-rated Working Alliance Inventory (WAI; Tracey and Kokotovic, 1989) after each session.

Figure 6 presents the evolution of L.'s and his therapist's ratings on the depth dimension of the SEQ, session after session. As we can see on the graph, L. provided flat and almost perfect scores after all the sessions. In contrast, his therapist's ratings were less inflated and show much more fluctuations. Their ratings were not correlated.

#### Patient Relationship Interview at Termination

It is very interesting to notice that when administered the termination relationship interview, L. reported being satisfied with therapy, and seemed quite realistic about the ability of a short-term therapy to promote change:

“Interviewer: Could you start by helping me to get oriented with your work with your therapist?

L.: Um well I um uh have had a lifelong problem with depression, I take medication I um, this was my fourth round of sessions with the [Name of the research program].

Interviewer: Oh, ok.

L.: And um well she was doing partly cognitive uh partly cognitive approach partly just relationship between patient and therapist and you know I really found it very helpful.

Interviewer: Mm-hm.

L.: It-it's not the answer to all my troubles but (pauses) I really found it to be helpful.”

When L. was asked about his response to the treatment termination, he provided an interesting and insightful response, as follows:

“L.: Well sh—(pauses) uh (shrugs) (pauses) I didn't cry or anything. I just (laughs) (pauses) I went on my way and then um (pauses) I think the next day (pauses) I had started having serious problems at work—

Interviewer: Mm-hm

L.: –they started to come up right after I ended therapy

Interviewer: How did your therapist respond when it was the end of the sessions?

L.: Uh (pauses) y'know she didn't act any differently than she normally acts, she kinda just went, “Nice working with you.” (pauses) She told me that if I wanted to ask her something ah y'know some sort of a—a longer term therapy—um that if I had any questions about that I could call her.

Interviewer: Are there any other separations that stand out in your mind?

L.: Well I've had a lot of separations. I separated with my wife, I uh I lost my parents, I um (pauses) …yeah I guess life is full of separations. (pauses) Um yeah (pauses) I had just um (pauses) well I when I (pauses) started um this guy I used to play with had recently committed suicide.

Interviewer: Mm.

L.: So I draw and uh this guy was an artist and he committed suicide and I had y'know strange feelings about that and (pauses) y'know that was a kind of separation. Uh, I don't I don't think that y'know, like she's the fourth therapist that I've had—I think that the last session is never really—quite easy.

Interviewer: Mm-hm

L.: I get too dependent on the therapists and um (pauses) I feel like I can't get along without help.”

L. also compared his therapist to his previous therapists at the same research program and concluded: “My previous therapist in this program I thought there were things that she didn't quite get about me, but I didn't feel that with [therapist's name].”

L.'s positive responses seem to contradict McLeod (2011) claim that patients tend to be more critical about their therapy when interviewed about it than when asked to report their symptoms on standardized symptom measures.

## Discussion

The findings generated by the different methods throughout this study convey a complex and nuanced picture of L.'s outcome at termination, and support two main interpretative frameworks which are not mutually exclusive: (1) The treatment may have been beneficial for L., in that it slowed down his naturally occurring path toward deterioration in psychological functioning; (2) The treatment may have failed to develop L.'s sense of agency, therefore culminating in a sudden deterioration, toward the end of treatment, triggered by termination.

First and foremost, the descriptive analysis, and to a lesser extent the 3RS coding suggest that L. did not have a strong sense of self-worth and tended to engage in avoidance strategies, such as passivity and procrastination, rather than confront his feelings and difficulties. L. had attended three brief treatments before the cognitive-behavioral therapy addressed in the present study, and had achieved only mild and transient progress, suggesting that he was resistant to change. Additionally, L.'s thought process seemed to be characterized by digressive and ruminatory processes. All together, these elements seem to suggest that L.'s chances to change within the context of a short-term therapy may have been limited. L.'s choice to repeat brief treatments rather than seek for long-term therapies more adapted to his needs may also suggest some ambivalence toward therapy and change. Last, L. experienced a series of life circumstances, such as the death of his friend, the changes at his workplace, concerns about his girlfriend's health, and most importantly, his own aging. Together with his difficulty confronting the realization that he may not become the artist he dreamt to be, these stressors probably put him on a worsening trajectory. The fact that L. did not reliably deteriorate throughout treatments 1–3 at the research program, though deteriorated by the end of treatment 4 and 5 may support this hypothesis. In these circumstances, it is not possible to determine with certainty what would have been L.'s trajectory had he not attended treatment, rendering it challenging to formulate definite statements about the therapy success.

Several findings nevertheless seem to suggest that the treatment may well have been beneficial. First, L.'s therapist rated L. as improved (though not reliably improved) in interpersonal functioning. Additionally, L.'s self-reported increased anxiety and depressive mood toward the end of treatment may be indicative of L.'s improved capacity to report his symptoms accurately because of his expanding self-awareness and insight. This explanation aligns with McLeod (2001) claim according to which patients' understanding of self-report questionnaires changes throughout treatment as a consequence of increased insight, so that they do not rate themselves on the same constructs at intake and termination. Last, the process variables examined suggest that the therapist factors discussed in the treatment failure literature, i.e., negative countertransference, rejection of the patient, or critical stance, were not factors in the present case. On the contrary, L.'s therapist proved to be very empathic, attuned, non-judgmental, and accepting of L. She was also optimistic and kept trying to engage him despite his tendency to fall back on repetitive and idiosyncratic story-telling. Her efforts indeed yielded fruit, as attested by L.'s compliance with homework. These findings seem to suggest that the treatment did not aggravate L.'s worsening trajectory and may in fact have minimized it.

On the other hand, the examination of the therapy process also suggests that the therapist's directive and pro-active style may have contributed to confine L. into a passive, avoidant and dependent position rather than encourage him to experience a more active and leading stance. The therapist's personal style was probably reinforced by the cognitive-behavioral orientation which guided her treatment and emphasized structured interventions, initiated by the therapist. Interventions aimed at fostering the development of L.'s own sense of agency and capacity for moment-to-moment self-awareness were not included in the therapist's repertoire. During his termination interview, L. indeed expressed his dependency toward his different therapists in the program, and his difficulty facing life challenges without their guidance. Accordingly, it is not unreasonable to hypothesize that L. may have experienced an actual, sudden worsening at the end of his treatment, caused by the termination, and different from a progressive deterioration occurring throughout treatment. In a similar vein, it is not unreasonable to postulate that therapy itself may have reinforced the maladaptive relational pattern that L. seems to have an inclination for. Namely, L.'s feelings of being safe and comfortable in his dependent and passive relationship with his therapist may have consolidated his core beliefs about his needs to be taken care of. Future research will need to gather outcome data session by session, rather than at intake and termination, in order to differentiate between deterioration throughout treatment and sudden deterioration possibly caused by termination anxiety.

Despite his deterioration on the symptom and interpersonal functioning measures, L. reported his satisfaction with therapy and the therapist, as well as his belief that the treatment was helpful. This finding is in line with the literature on patients' satisfaction with treatment, according to which patients' self-reported change is not correlated with satisfaction (Lunnen and Ogles, 1998). This finding, indisputably illustrated in L.s' case, may in fact suggest that the emotional experience provided by therapy, and its potential ability to increase one's feelings of acceptance and well-being probably need to be considered as outcome, beyond one's actual symptomatic and interpersonal change.

## Conclusions and Future Directions

This case-study illustrated Dimidjian and Hollon's taxonomy of outcome (2010) as well as Wampold and Imel's conceptualization of deterioration vs. harm. According to the former, patients' change in symptomatology is not indicative of treatment success when taken independently from patients' expected course of disease. Indeed, L.'s background, his personality style, and his successive treatment outcomes suggest that his symptoms may have deteriorated more severely had he not participated in treatment. For the later, deterioration in functioning throughout treatment can be caused by a variety of factors that are not related to the treatment *per se*. L.'s deterioration was indeed not induced by the treatment itself, so that his therapy cannot be qualified as harmful. Additionally, L.'s case emphasized that human functioning and its evolution throughout therapy are multi-faceted and difficult to assess in a comprehensive and fully reliable manner using quantitative methods only: L. indeed reliably deteriorated on two measures of outcome, and yet he felt satisfied with his therapy, improved his capacity for self-awareness, experienced a warm and productive relationship, and learned more adaptive ways of thinking and handling life challenges. Last, the present study suggests that the combination of client factors such as avoidance and lack of agency, therapist factors such as directiveness, and therapy factors such as brief treatment and high structure, may have played a role in L.'s sudden relapse and deterioration at termination. Future research may help clarify the extent to which this combination of factors indeed increases the likelihood of sudden deterioration at termination.

## Ethics Statement

The study was performed with archival data collected at the Brief Psychotherapy Research Program, New York, approved by the Icahn school of Medicine at Mount Sinai IRB, application #048–88.

## Author Contributions

SB-E performed the analyses and completed the writing of the manuscript. CE performed the analyses related to the 3RS system and wrote the section on that topic. LK collaborated with the first author to interpret the findings and revise successive drafts of the manuscript. BG supervised the statistical analyses involving all the quantitative measures and revised the successive drafts of the manuscript. JM is the PI of the research program in which this study took place. He was responsible for the data collection and guided SB-E throughout all the steps leading to the completion of the manuscript.

### Conflict of Interest Statement

The authors declare that the research was conducted in the absence of any commercial or financial relationships that could be construed as a potential conflict of interest.
